# A novel T-cell exhaustion-related feature can accurately predict the prognosis of OC patients

**DOI:** 10.3389/fphar.2023.1192777

**Published:** 2023-05-22

**Authors:** Kemiao Yuan, Songyun Zhao, Bicheng Ye, Qi Wang, Yuan Liu, Pengpeng Zhang, Jiaheng Xie, Hao Chi, Yu Chen, Chao Cheng, Jinhui Liu

**Affiliations:** ^1^ Department of Oncology, Traditional Chinese Medicine Hospital of Wuxi, Wuxi, China; ^2^ Department of Neurosurgery, Wuxi People’s Hospital Affiliated to Nanjing Medical University, Wuxi, China; ^3^ School of Clinical Medicine, Yangzhou Polytechnic College, Yangzhou, China; ^4^ Department of Gastroenterology, Affiliated Hospital of Jiangsu University, Zhenjiang, China; ^5^ Department of General Surgery, Wuxi People’s Hospital Affiliated to Nanjing Medical University, Wuxi, China; ^6^ The First Affiliated Hospital of Nanjing Medical University, Nanjing, China; ^7^ Southwest Medical University, Luzhou, China; ^8^ Wuxi Maternal and Child Health Care Hospital, Wuxi, China; ^9^ Department of Gynecology, The First Affiliated Hospital of Nanjing Medical University, Nanjing, China

**Keywords:** t cell exhaustion, OC, ScRNA-seq, machine learning, immunotherapy

## Abstract

The phenomenon of T Cell exhaustion (TEX) entails a progressive deterioration in the functionality of T cells within the immune system during prolonged conflicts with chronic infections or tumors. In the context of ovarian cancer immunotherapy, the development, and outcome of treatment are closely linked to T-cell exhaustion. Hence, gaining an in-depth understanding of the features of TEX within the immune microenvironment of ovarian cancer is of paramount importance for the management of OC patients. To this end, we leveraged single-cell RNA data from OC to perform clustering and identify T-cell marker genes utilizing the Unified Modal Approximation and Projection (UMAP) approach. Through GSVA and WGCNA in bulk RNA-seq data, we identified 185 TEX-related genes (TEXRGs). Subsequently, we transformed ten machine learning algorithms into 80 combinations and selected the most optimal one to construct TEX-related prognostic features (TEXRPS) based on the mean C-index of the three OC cohorts. In addition, we explored the disparities in clinicopathological features, mutational status, immune cell infiltration, and immunotherapy efficacy between the high-risk (HR) and low-risk (LR) groups. Upon the integration of clinicopathological features, TEXRPS displayed robust predictive power. Notably, patients in the LR group exhibited a superior prognosis, higher tumor mutational load (TMB), greater immune cell infiltration abundance, and enhanced sensitivity to immunotherapy. Lastly, we verified the differential expression of the model gene CD44 using qRT-PCR. In conclusion, our study offers a valuable tool to guide clinical management and targeted therapy of OC.

## Introduction

Ovarian cancer is a prevalent gynecological malignancy and ranks sixth as the leading cause of cancer-related death in women (Bronger et al., 2016), with approximately 150,000 women losing their lives each year (Sung et al., 2021). Unfortunately, due to ineffective screening methods and unnoticeable early symptoms, more than 75% of patients are diagnosed with an advanced stage, and over 70% experience recurrence after treatment (Dinh et al., 2008). Despite progress in treatment strategies and technologies, ovarian cancer mortality rates still remain high (Hubbell et al., 2021). Therefore, developing novel multigene-based models for diagnosing and predicting ovarian cancer prognosis is crucial, considering the complex molecular mechanisms that affect it and the lower precision of individual gene prediction models.

Upon encountering a pathogen, initial T cells respond by proliferating towards effector and memory T cells in response to antigen, costimulatory signals, and inflammation (Wherry, 2011). However, exhausted T cells lose their ability to respond to additional proliferative signals and become unresponsive to future stimulation with the same antigen, resulting in a loss of memory homeostasis (Doering et al., 2012; Chow et al., 2022). Furthermore, these cells also lose their ability to respond to additional proliferative signals (Moskophidis et al., 1993; Doering et al., 2012). Recent research has demonstrated that blocking co-inhibitory receptors on the surface of exhausted CD8^+^ T cells, such as PD-1, reactivates the cytolytic effect of T cells (Day et al., 2006; Wherry and Kurachi, 2015). Nevertheless, the mechanism behind immune checkpoint blockade (ICB) and T-cell exhaustion requires further investigation since T-cell exhaustion plays a crucial role in immune dysfunction and immune escape in cancer patients.

Identifying tumor immune profiles and immune characteristics of patients with different tumors is crucial, and the tumor immune microenvironment has been highlighted as a crucial factor in cancer progression and treatment response (Zhao et al., 2022a). Although tumor-infiltrating T cells are critical in recognizing and killing tumor cells, most infiltrating T cells become ‘exhausted’ due to the level and number of expressed inhibitory receptors (IRS) (McLane et al., 2019). High-grade ovarian cancer has a tumor immunosuppressive environment with a high proportion of Tex and regulatory T cells (Treg), resulting in the exhaustion of specific tumor-infiltrating lymphocytes (TILs) and interaction with tumor antigens (Yang et al., 2022).

In addition, there is evidence to suggest that T-cell exhaustion is a continuous and evolving process (Philip et al., 2017). As such, the objective of this research is to detect and describe patients with distinct T-cell exhaustion patterns. This study will employ bulk sequencing and single-cell RNA sequencing (scRNA-seq) data from ovarian cancer to pinpoint TEX-associated genes (TEXRGs) with significant prognostic value. To examine the prognostic impact of TEXRGs on the progression and prognosis of ovarian cancer, a TEX-related prognostic signature (TEXRPS) was developed using a comprehensive machine-learning combination.

## Materials and methods

Source of raw data To conduct this study, 10 × scRNA-seq data from GSE154600, which included 5 samples of ovarian cancer (OC) and a total of 52,384 cells, were employed. Additionally, gene expression profiles, measured in fragments per kilobase million (FPKM), and clinicopathological data for OC were sourced from The Cancer Genome Atlas (TCGA) and the Gene Expression Omnibus (GEO) databases. Further analysis was carried out on two GEO cohorts (GSE9891, GSE63885) and the TCGA-OV cohort. To ensure consistency in transcript quantification, FPKM was converted to transcripts per million (TPM), which were deemed equivalent to the GEO microarray transcripts (Conesa et al., 2016). Subsequently, the “affy” R package was utilized for background calibration, normalization, and log2 transformation on all GEO raw datasets (Irizarry et al., 2003). The “sva” R package was then used to remove batch effects, and patients lacking survival information were excluded (Leek et al., 2012). In total, 711 patients were included in the analysis, consisting of 360 patients from the TCGA cohort, 276 patients from the GSE989 cohort, and 75 patients from the GSE63885 cohort.

### Processing of single-cell sequencing data

Our approach to analyzing ovarian cancer single-cell sequencing data involved several key steps. First, we conducted quality control (QC) by assessing the proportion of mitochondrial or ribosomal genes. Next, we used the “Seurat” R package (Macosko et al., 2015)to convert the 10 × scRNA-seq data into Seurat objects, identifying the first 2,000 highly variable genes with the “FindVariableFeatures” function. To reduce feature dimensionality while preserving data integrity, we applied principal component analysis (PCA) and unified flow approximation and projection (UMAP) to an additional 2,000 genes to identify distinct cell subpopulations. We then used the “FindAllMarkers” tool to detect marker genes in different clusters, setting cut-off values for both |log 2 FC| and min pct to 0.25. To annotate different cell types, we employed the “Singler” R package (Aran et al., 2019). Additionally, we performed enrichment analysis using the “analyze_sc_clusters” function of the “ReactomeGSA” R package (Griss et al., 2020). For pseudotime analysis of scRNA-seq, a powerful tool to understand the sequence of gene expression changes during a cell state transition, we relied on the classic “Monocle” R package (Qiu et al., 2017; Chi et al., 2023). Finally, we conducted intercellular communication analysis and network visualization using both the “CellChat” (Jin et al., 2021) and “patchwork” R packages.

### WGCNA and scoring of TEX pathway

WGCNA, or Weighted Gene Co-expression Network Analysis, is a technique that identifies co-expressed gene modules and explores the correlation between the gene network and the phenotype of interest, as well as the hub gene in the network (Langfelder and Horvath, 2008). In this study, we utilized a TEX signaling pathway study (Zhang et al., 2022) and its corresponding marker genome from the Molecular Signaling Database (MSigDB, V7.2) to estimate the activity score of the TEX pathway for each patient. We used the “GSVA” R package (Hanzelmann et al., 2013) to perform this analysis, and the resulting TEX pathway activity scores for each patient are listed in Supplementary Table S1.

### Establishment of TEX-related prognostic signature in ovarian cancer

We integrated and analyzed the expression profiles of TCGA, GSE9891, and GSE63885 cohorts to identify TEX-related prognostic signatures (TEXRPS) using a novel computational framework that employed a combination of machine learning algorithms (MLAs). The candidate TEX-related genes (TEXRGs) were obtained using the Weighted Gene Co-expression Network Analysis (WGCNA). Subsequently, the TEXRGs from the TCGA-OV dataset with prognostic potential were evaluated using univariate Cox regression analysis with a *p*-value threshold of 0.2. To enhance the reliability of our results, we performed 10-fold cross-validation and utilized a total of 80 combinations of 10 machine learning algorithms (MLAs), which included Lasso, Ridge, supervised principal components (SuperPC), stepwise Cox, generalized boosted regression modeling (GBM), CoxBoost, stochastic survival forest (RSF), elastic network (Enet), partial least squares regression for Cox (plsRcox), and survival support vector machine (survival-SVM).

To create the TEXRPS, a combination of RSF and CoxBoost algorithms was utilized. The CoxBoost algorithm was employed to filter the most valuable TEXRGs, while the RSF algorithm was used to derive the most trustworthy models. Subsequently, the RSF algorithm was employed to filter the most reliable model. A log-rank score test was conducted for splitting survival trees as previously described (Hothorn and Lausen, 2003). Initially, the x-variable x was assumed to be ordered as x1 ≤ x2 ≤ … ≤ xn, and the ranks for each survival time Tj (j ∈ [1, … , n]) were computed using the following equation:

$$a_{j}=\delta_{j}-\sum_{k=1}^{\Gamma_{j}}\frac{\delta_{k}}{n-\Gamma_{k}+1}$$

where *Γ*_*j*_ represents the index of the order for T_j_ and *Γ*_*k*_ = #[t: Tt ≤ T_k_]. The log-rank score test was performed as follows:

$$Risk\;score=S\left(x,c\right)=\frac{\underset{xk\leq c}{\sum }\left(a_{j}-n_{l}\bar{a}\right)}{\sqrt{n_{l}\left(1-\frac{n_{l}}{n}\right)s_{a}^{2}}}$$

Where $s_{a}^{2}$ and $\bar{a}$ represent the sample variance of [a_j_: j = 1, . . ., n] and sample mean, respectively. The log-rank score splitting by |S (x, c) | was used to determine the measure of node separation. The best split is reached by maximizing this value over x and c.

To assess the potential of TEXRPS as an independent prognostic indicator for patients with OC, both univariate and multivariate Cox regression analyses were carried out. Furthermore, a nomogram was constructed using the “rms” R package to predict the OS of clinical patients at 1, 3, and 5 years based on age, grade, stage, and risk grouping. The accuracy of the nomogram predictions was verified by calibration analysis (Zhao et al., 2023a).

### Somatic mutation analysis

The somatic variant data in mutation annotation format (MAF) was analyzed using maftools to examine the mutation data from OC samples (Mayakonda et al., 2018). The tumor mutation burden (TMB) score was calculated for each patient with OC, and its correlation with the risk score was examined. To calculate the TMB score, the total mutations were divided by the total covered bases and then multiplied by 10^6^ (Robinson et al., 2017; Zhao et al., 2022a). Additionally, the prognostic significance of TMB in OC was evaluated using Kaplan-Meier analysis.

### Immune microenvironment

The “ssGSEA” R script was used to determine the relative proportions of infiltrating immune cells. Additionally, TME scores were calculated using the “ESTIMATE” R package, which provided stromal, immune, and estimated scores for both groups. The gene sets related to cancer and immunity was obtained from Xu et al.’s website (Xu et al., 2018) (http://biocc.hrbmu.edu.cn/TIP/) and a set of genes positively associated with antiPD-L1 drug response from Mariathasan’s study features (Mariathasan et al., 2018).

### Immunotherapy prediction and chemotherapy sensitivity analysis

The Cancer Immunome Atlas (TCIA) web tool facilitates comprehensive immunogenomic analysis and generates a quantitative tumor immunogenicity score, termed the Immunophenotype Score (IPS), ranging from 0 to 10, which can serve as a predictive marker for the response to immune checkpoint inhibitors (ICIs) (Charoentong et al., 2017). To evaluate the efficacy of chemotherapy and molecular drugs, we calculated the half-maximal inhibitory concentrations (IC50) for HR and LR subgroup samples using the “pRRophetic” R package. Moreover, the HPA database, encompassing proteomic, transcriptomic, and systems biology data, is capable of annotating various tissues, cells, and organs, among others. To confirm the expression profiles of TEXRGs, we performed immunohistochemistry on patient samples sourced from the HPA database.

### Immunohistochemical analysis and qRT-PCR

Cell lines including the ovarian epithelial cell IOSE, ovarian cancer SKOV-3, and A2780 were procured from the esteemed Shanghai Institutes for Life Sciences, affiliated with the Chinese Academy of Sciences in Shanghai, China. These cell lines were propagated in Roswell Park Memorial Institute 1640 medium fortified with 10% heat-inactivated fetal bovine serum, penicillin (10 U/mL), and streptomycin (50 μg/mL) under a 5% CO2 atmosphere at 37°C. RNA was extracted from both cells and tissues utilizing Trizol reagent (Invitrogen), followed by reverse transcription using SuperScript II reverse transcriptase (Invitrogen) in accordance with the manufacturer’s recommended protocol. Thereafter, the relative mRNA expression levels of CD44 and GAPDH (as a normalized control) were quantified by SYBR Premix Ex Taq II (Takara, Dalian. China). The primer sequences utilized for this purpose are as follows:

CD44:forward5″-CTGCCGCTTTGCAGGTGTA-3"; reverse5″-CATTGTGGGCAAGGTGCTATT-3″. GAPDHforward:5″-GGAGCGAGATCCCTCCAAAAT-3″; reverse5″-GGCTGTTGTCATACTTCTCATGG-3″.

### Statistical analysis

The R4.1.1 and its complementary package were utilized for all statistical analyses. To determine prognostic values and compare patient survival among different subgroups within each dataset, Kaplan-Meier survival analysis, and log-rank tests were employed (Zhao et al., 2022b). The difference between two normally distributed groups was assessed using the Student’s t-test, while the Wilcoxon test was utilized to compare two non-normally distributed variables. Multiple group comparisons were performed using the Kruskal–Wallis test as a non-parametric approach. Correlation coefficients were examined by Spearman’s correlation analysis. A *p*-value less than 0.05 was considered statistically significant for all statistical tests conducted.

## Results

### ScRNA-seq analysis of OC samples

**FIGURE 1:**
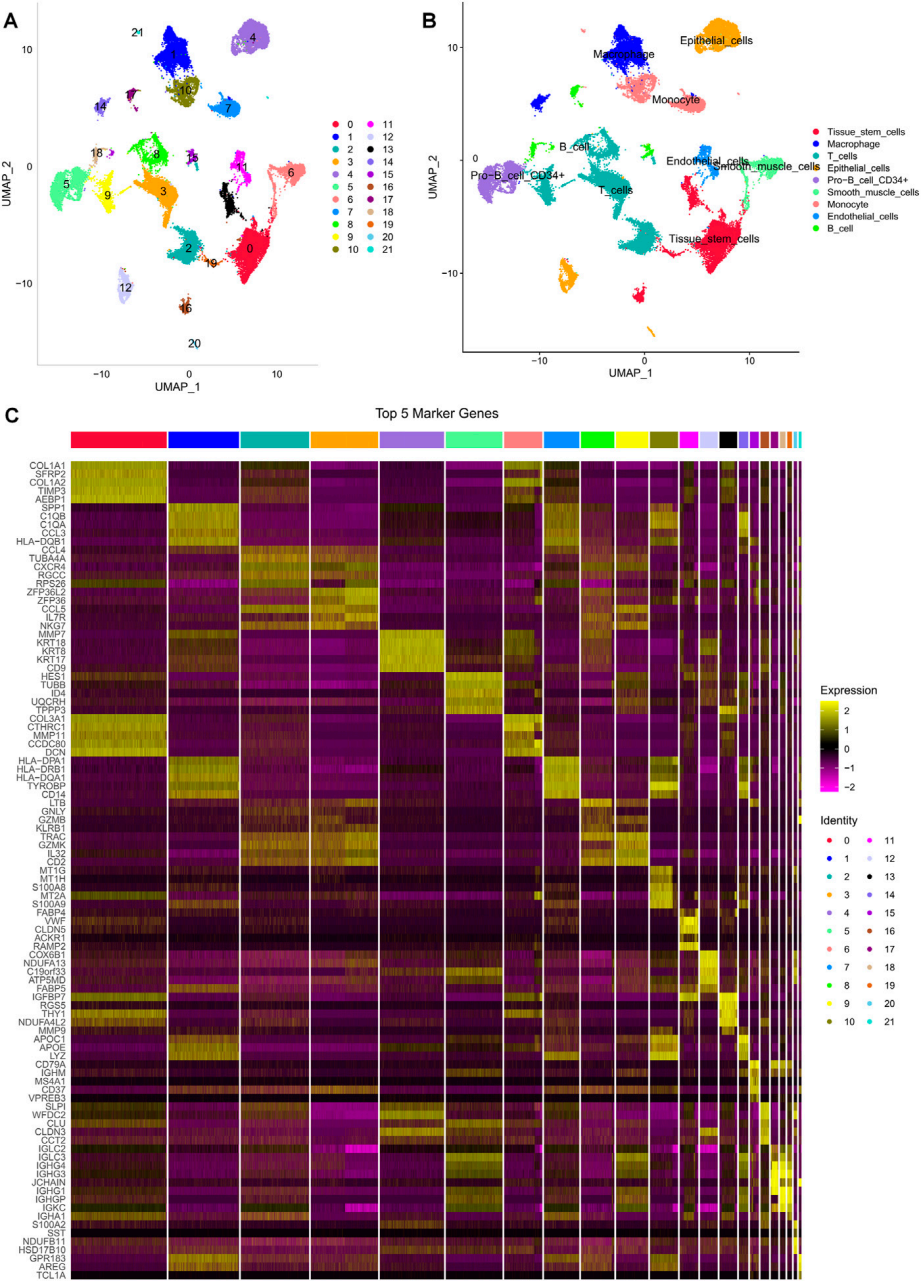
Different cell clustering in ovarian cancer 10 × scRNA-seq data. **(A,B)** Cluster annotation and **cell** type identification by UMAP. **(C)** Heat map of marker genes for different cell types.

We retrieved 10 × scRNA-seq data with 5 OC samples from the GSE154600 dataset. The initial cell counts before QC were 16,258, 16,662, 8,125, 5,644, and 4,795, respectively, and the QC process yielded 11,730, 13,469, 6,199, 4,534, and 3,747 cells, respectively (Supplementary Figure S1A). We depicted the top 2000 highly variable genes in Supplementary Figure S1B. PCA and UMAP analysis were performed to preprocess the high-dimensional feature data, revealing 21 distinct cell subpopulations (Figure 1A). We applied the “SingleR” R package to classify and visualize the cell types and discovered 9 significant cell types, including monocytes, macrophages, tissue stem cells, epithelial cells (tumor cells), endothelial cells, smooth muscle cells, primitive cells, B cells, and T cells (Figure 1B). The top 5 marker gene expressions for the different cell subpopulations are shown in Figure 1C.

**FIGURE 2:**
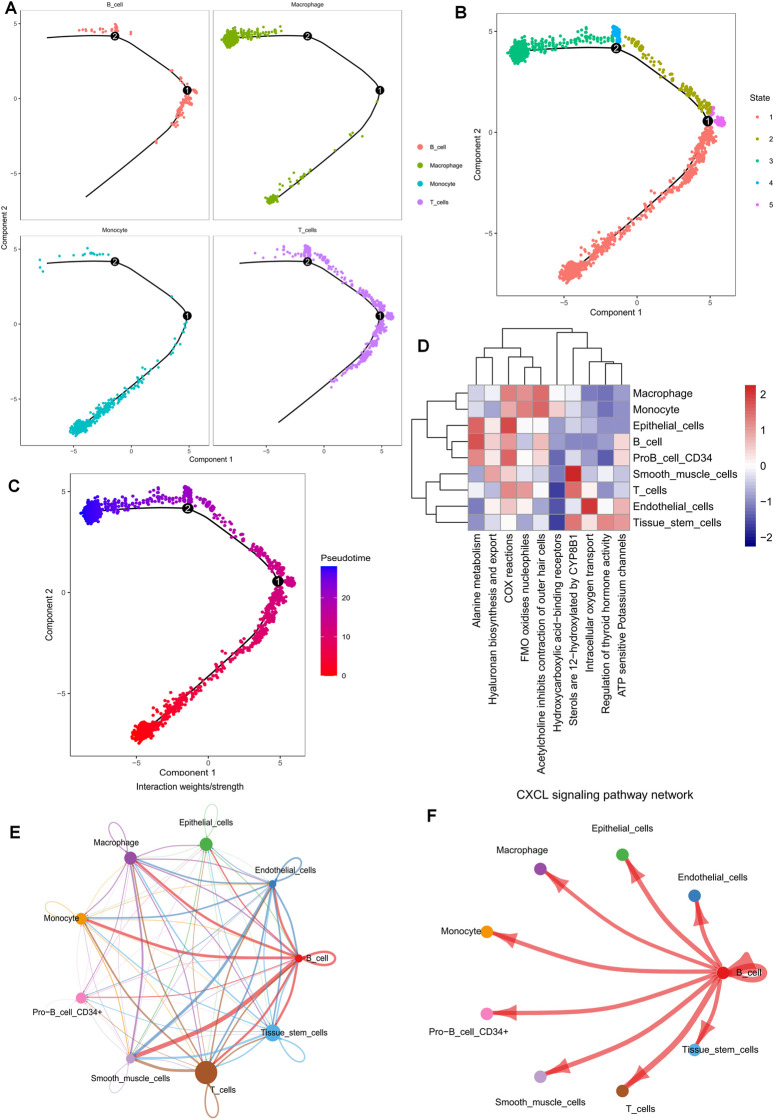
Pseudotime time series analysis, enrichment analysis, and cell communication analysis of 10 × scRNA-seq data from ovarian cancer. **(A–C)** Cell trajectory analysis and pseudotime of identified immune cell types. **(D)** Functional enrichment analysis of all cell types using the “ReactomeGSA” software package. **(E,F)** Cellular communication networks were inferred by calculating the likelihood of communication. Studies of intercellular communication networks suggest that the CXCL signaling pathway plays an important role in intercellular communication networks.

To determine the cell trajectory and pseudotime of the four immune cells, we used the “monocle” R package and discovered that T cells were predominantly associated with states 2, 3, and 5 and resided in the middle of cell developmental time (Figures 2A–C). “ReactomeGSA” functional enrichment analysis revealed that T cells and smooth muscle cells were significantly involved in the sterol hydroxylation pathway (Figure 2D). By examining the communication likelihood, we analyzed the cell-cell communication network (Figure 2E, Supplementary Figure S2A). CXCL receptor signaling pathway plays a crucial role in regulating the tumor microenvironment, autoimmune diseases, infections, and fibrosis, and CXCL has been a major focus of pharmaceutical research and development to enhance the efficacy of tumor immunotherapy (Daniel et al., 2020). Therefore, we inferred the cellular communication networks based on specific pathways and ligand receptors and found that the CXCL signaling pathway is crucial in the T and B Cell communication network (Figure 2F, Supplementary Figure S2B).

### Identification of candidate TEX-related genes

**FIGURE 3:**
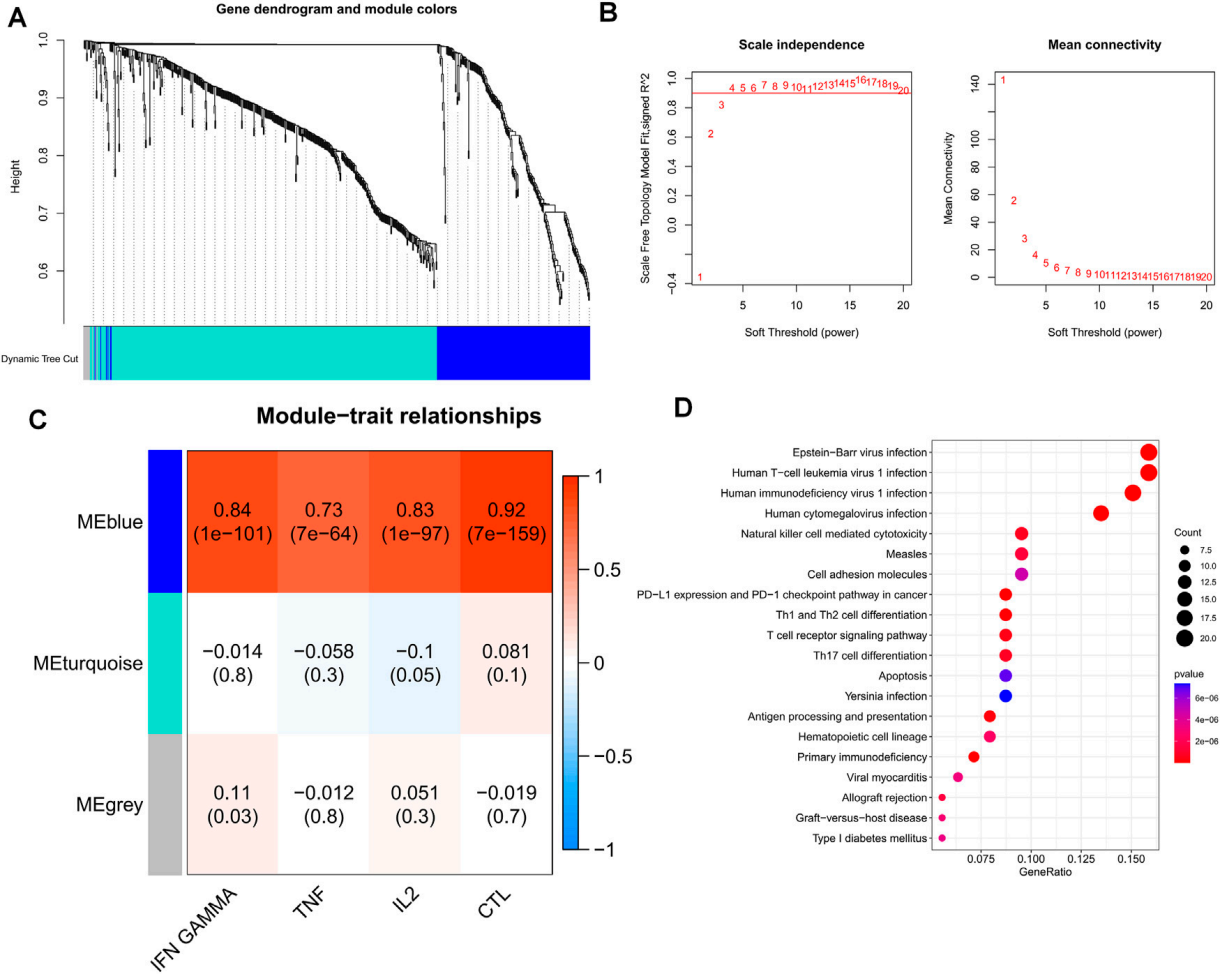
Identification of candidate TEXRGs in the TCGA-OV cohort. **(A)** Cluster dendrogram. **(B)** Scale independence and average connectivity. **(C)** Heat map of correlations between TEX pathways and modules. **(D)** Results of KEGG enrichment analysis of candidate TEXRGs.

Drawing on prior research, we computed GSVA enrichment scores for four pathways linked to T-cell exhaustion for each TCGA-OV sample. We then identified 627 marker genes from T cells and incorporated them into WGCNA to pinpoint the key modules that were most pertinent to the advancement of T-cell exhaustion in the TCGA cohort. While constructing the co-expression network, we observed that the soft threshold power β reached a value of 4 with a scale-free topology fit index of 0.9 (Figure 3B). Afterward, we applied the ‘merged dynamics’ algorithm to generate three modules (Figure 3A). After scrutinizing correlation coefficients and *p*-values, we discerned that the blue module exhibited the strongest correlation with scores linked to T-cell exhaustion progression (Figure 3C); as a result, we designated the blue module as the pivotal module. Our KEGG enrichment analysis indicated that 195 TEXRGs from the blue module were notably associated with T-cell leukemia virus infection, as well as pathways related to immunodeficiency virus infection (Figure 3D).

### Construction of TEXRPS

**FIGURE 4:**
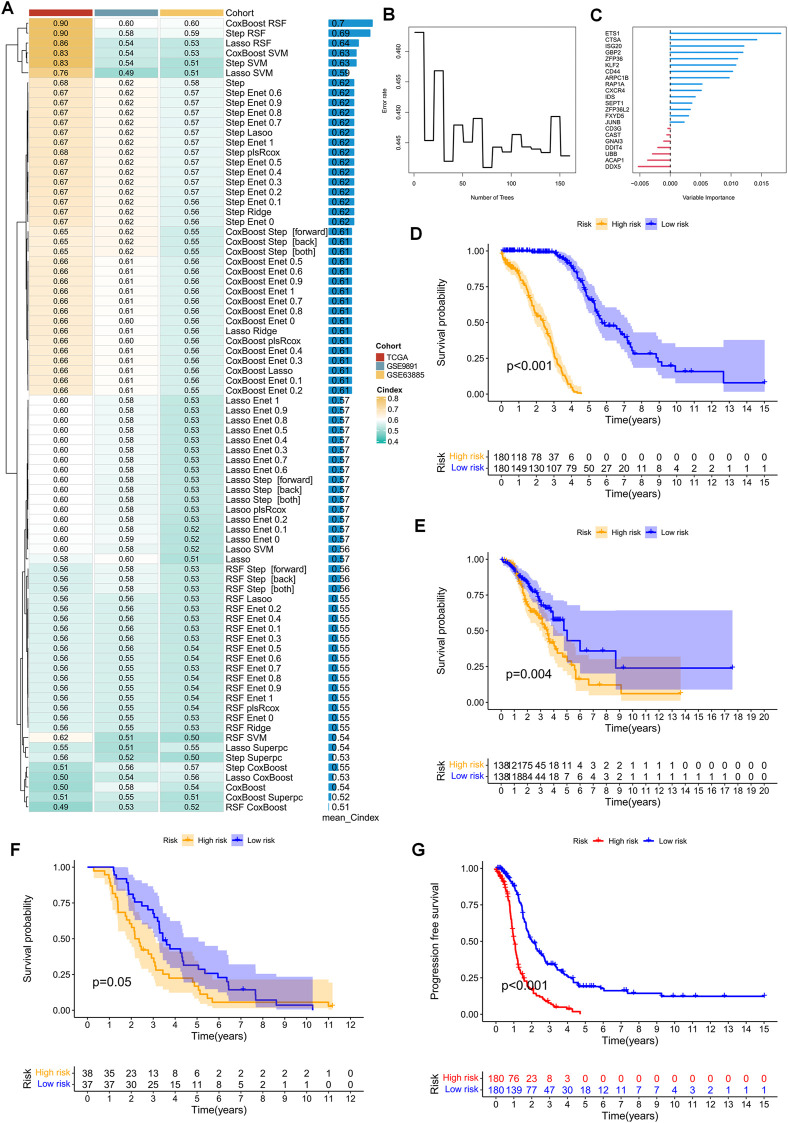
TEX-related prognostic signature and its prognostic value constructed by machine learning-based integration. **(A)** Eighty machine learning-based integration prediction models were fitted with 10-fold cross-validation. The C-index was calculated for each model in the training and validation cohorts, including the TCGA-OV, GSE9891, and GSE63885 cohorts. **(B)** The number of trees is determined by minimal error. **(C)** Importance of the 22 most valuable genes based on the RSF algorithm. **(D–F)** Kaplan-Meier survival curves for OS in patients in the HR and LR groups in the TCGA-OV, GSE9891, and GSE63885 cohorts. **(G)** Kaplan-Meier survival curves for PFS in patients in the HR and LR groups in the TCGA-OV cohort.

The TCGA-OV cohort, consisting of 376 patients, served as the training cohort. Initially, 47 prognostic genes were identified via univariate Cox analysis, with a cut-off *p*-value of 0.2, and were subsequently used as seed genes in our machine learning-based integrative procedure, which aimed to construct a prognostic signature for TEX-related diseases. To evaluate the predictive performance of 80 machine learning-based integrative models, 10-fold cross-validation was conducted in the training cohort, where the C-index was computed for each model across all datasets. Notably, the evaluation of a model’s performance not only relied on its robustness in the training cohort but also its performance in the validation cohort. Remarkably, the most superior model comprised a combination of CoxBoost and Random Survival Forest (RSF), which yielded an average C-index of 0.7, the only model with such a high C-index (Figure 4A). Accordingly, the optimal TEX-related prognostic signature was developed based on the combination of the CoxBoost and RSF algorithms, where the former identified 22 TEX-related genes (Supplementary Table S2) and the latter was responsible for constructing the most reliable prognostic model (Figures 4B, C). Subsequently, using the above procedure, the risk score for each patient was computed. Based on the median risk score of the training cohort, the patients were classified into two groups: high-risk (HR) and low-risk (LR). Strikingly, the LR group displayed better overall survival (OS) than the HR group in the TCGA cohort (Figure 4D). Additionally, the prognostic significance of TEXRPS was confirmed by adopting the same cut-off values obtained from the GEO training cohorts. For the GSE9891 and GSE63885 cohorts, patients classified into the LR group exhibited a superior OS (Figures 4E, F). Notably, in the TCGA cohort, the LR group also demonstrated better progression-free survival (PFS) than the HR group (Figure 4G).

### Establishment of prognostic nomograms and validation of clinical features

**FIGURE 5:**
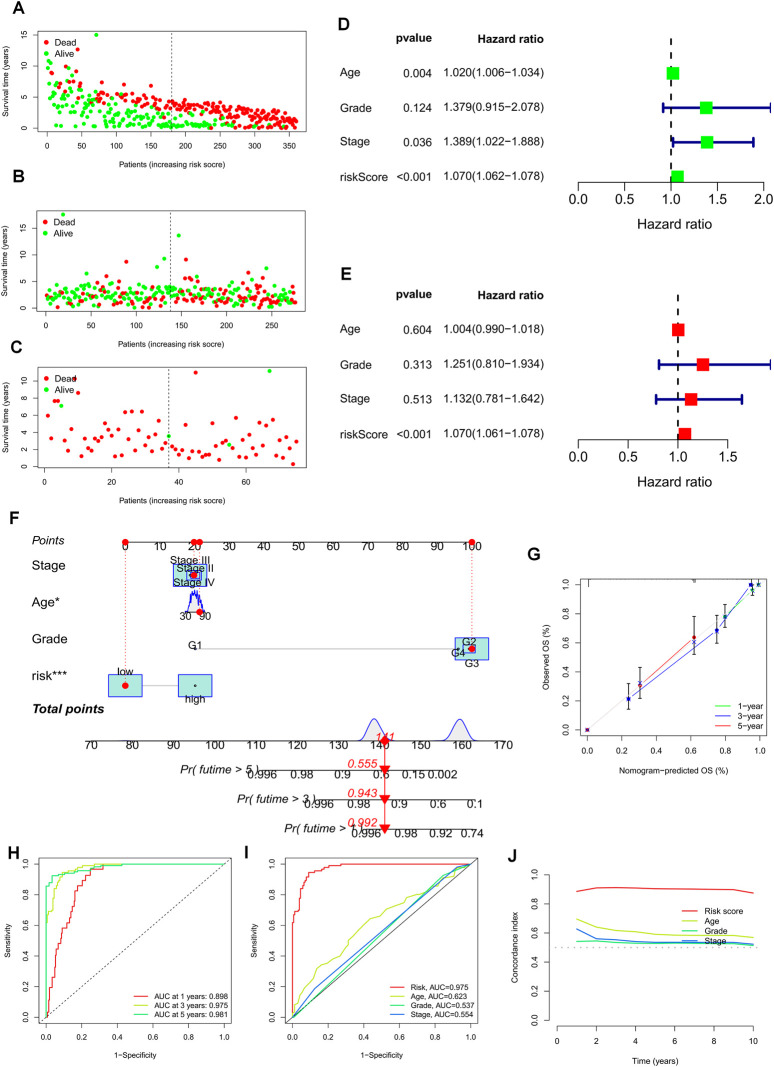
Prognostic value of risk scores and clinical characteristics of OC patients. **(A–C)** Risk maps were used to illustrate the survival status of each sample in the TCGA-OV, GSE9891, and GSE63885 cohorts. **(D)** Univariate and **(E)** multivariate COX analysis to assess prognostic characteristics and clinical features (including age, grade, and stage). **(F)** Nomogram of risk groupings and clinical characteristics predicting survival at 1, 3, and 5 years. **(G)** Calibration curves tested for agreement between actual and predicted outcomes at 1 year, 3 years, and 5 years. **(H)** AUC values for the TCGA cohort risk groupings at 1, 3, and 5 years. **(I)** AUC values for TCGA cohort risk subgroups and clinical characteristics at 3 years. **(J)** Concordance index (C-index) for the TCGA cohort. *p < 0.05; **p < 0.01; ***p < 0.001.

The risk plots for the TCGA-OV, GSE9891, and GSE63885 cohorts illustrate the individual survival outcomes of each patient, with higher risk scores associated with worse prognostic outcomes (Figures 5A–C). Through univariate and multivariate Cox analysis of the TCGA and two GEO validation cohorts, we determined that risk scores could serve as an independent prognostic factor for patients, even when compared to other common clinical characteristics (Figures 5D, E, Supplementary Figures S3A–D). To enhance the clinical utility of the risk model, we integrated a risk regression model based on the TCGA cohort, age, grade, and stage into a nomogram for predicting the overall survival of OC patients (Figure 5F). Notably, risk scores demonstrated greater prognostic accuracy compared to other clinical characteristics. Our results suggest that utilizing a risk model based on 22 TEXRGs can improve the accuracy of OC patient prognosis. The calibration curves demonstrated good agreement between predicted and observed values at 1, 3, and 5 years (Figure 5G). In the TCGA cohort, our constructed TEXRPS exhibited excellent performance in predicting OS for patients (AUCs for 1-, 3-, and 5-year OS: 0.898, 0.975, and 0.981) (Figure 5H). The area under the curve for the 3-year risk score was significantly higher than for other clinical features (Figure 5I). Additionally, the risk score C-index was much greater than for other clinical features (Figure 5J). Our findings indicate that risk scores remain a reliable prognostic indicator in the GSE9891 and GSE63885 cohorts (Supplementary Figures S3E–J).

**FIGURE 6:**
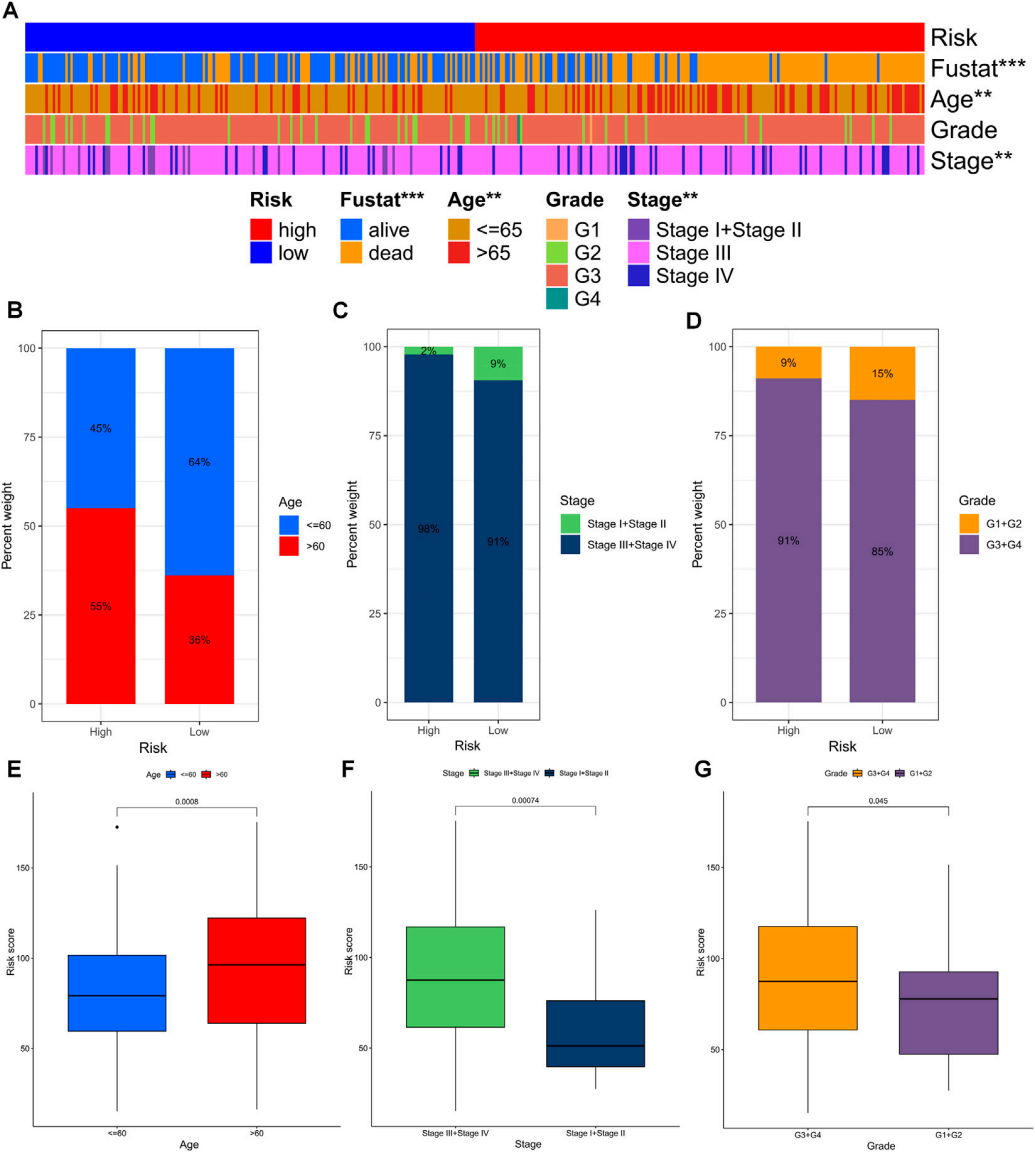
Distribution of risk scores in different clinical subtypes. **(A)** Bar charts of clinical characteristics associated with risk subgroups by chi-square test. **(B–D)** The proportion of patients in different clinical subtypes (age, grade, stage) in the HR and LR groups. **(E–G)** Differences in risk scores across clinical subtypes. **p < 0.01, ***p < 0.001.

The chi-square test revealed that the risk grouping was significantly associated with only three clinical characteristics, including survival status, age, and stage of the patients (Figure 6A). To further investigate and compare the differences in clinical characteristics among different risk groups for OS, we stratified OC patients into three subgroups based on age (≤60 and >60), pathological stage (I-II and III-IV), and pathological grade (G1-2 and G3-4). Notably, patients with lower risk scores had clear advantages in all subgroups, suggesting that TEXRPS is a dependable clinical prediction tool (Figures 6B–G). These results provide additional support for the reliability of our model.

### TMB analysis and survival analysis of TMB

**FIGURE 7:**
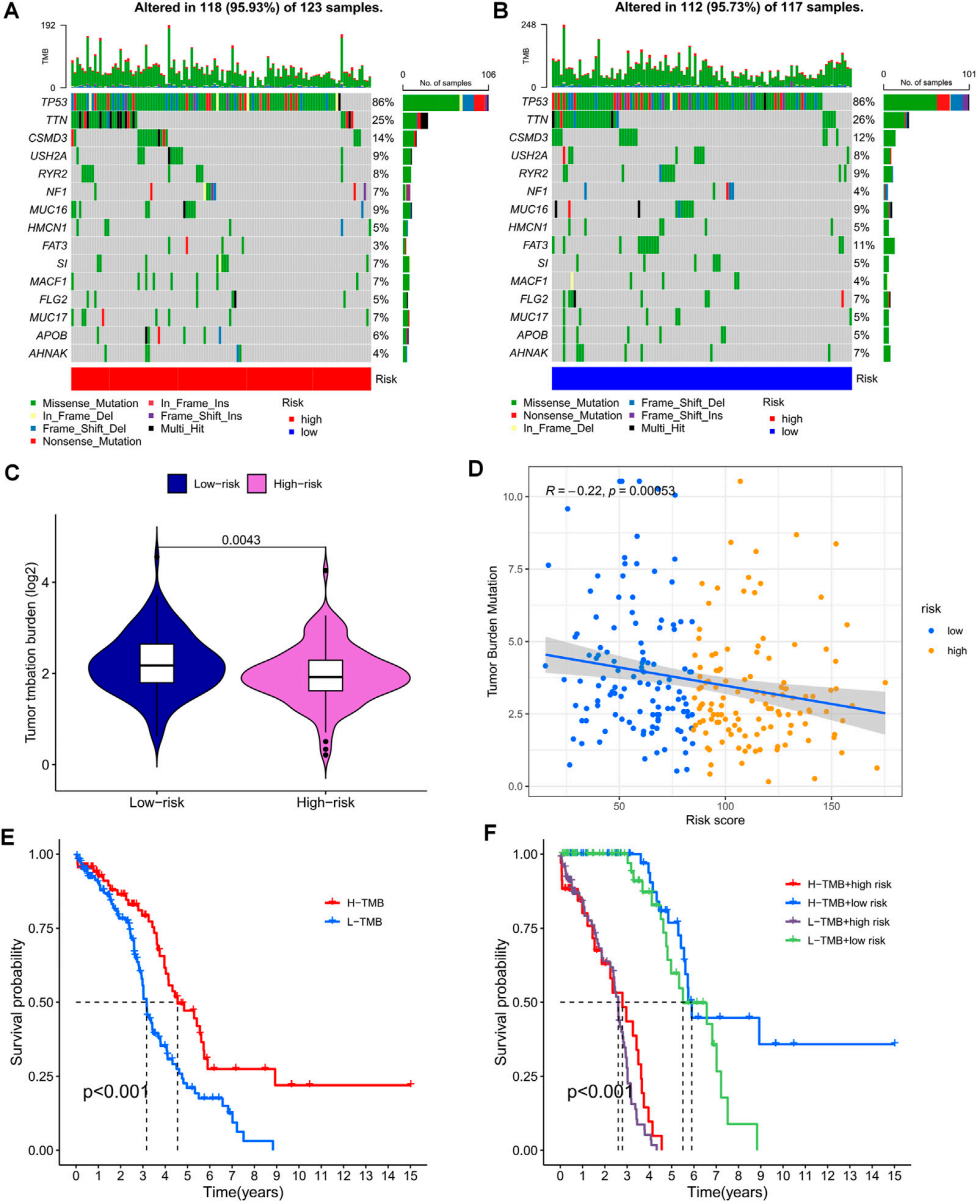
Mutation analysis based on risk score models. **(A,B)** Waterfall plots summarizing mutations in patients in the HR and LR groups. **(C)** Differences in tumor mutational load between the HR and LR groups. **(D)** Correlation between risk score and tumor mutation load. **(E)** Kaplan-Meier curves for the high and low TMB groups. **(F)** Kaplan-Meier curves for combined risk scores and TMB groupings.

Gene mutations are widely known to play a crucial role in tumorigenesis. Using the TCGA database, we utilized TEXRPS to visualize and correlate somatic mutation data, revealing TP53, TTN, and CSMD3 as the three most frequently mutated genes in both the HR and LR groups (Figures 7A, B). Variations in mutation status and expression patterns can have a significant impact on the immune response and subsequent clinical outcomes. To explore this relationship, we performed TMB analysis and found a marked difference between the two groups (*p* = 0.0043), with the LR group exhibiting higher TMB (Figure 7C). Interestingly, risk scores were strongly and negatively correlated with TMB (Figure 7D). Moreover, Kaplan-Meier survival analysis based on median TMB values divided into high and low TMB groups showed a better prognosis in the high TMB group (*p* < 0.001). This finding suggests that TMB may serve as an indicator of poor prognosis in OC patients (Figure 7E). Significantly, the combined use of risk scores and TMB to stratify patients into four groups for survival analysis highlighted the high TMB and LR groups as having the best prognosis (*p* < 0.001). These results further validate the predictive capability of the model and identify the optimal prognostic subgroup for clinical application (Figure 7F).

### The tumor microenvironment and immune cell infiltration

**FIGURE 8:**
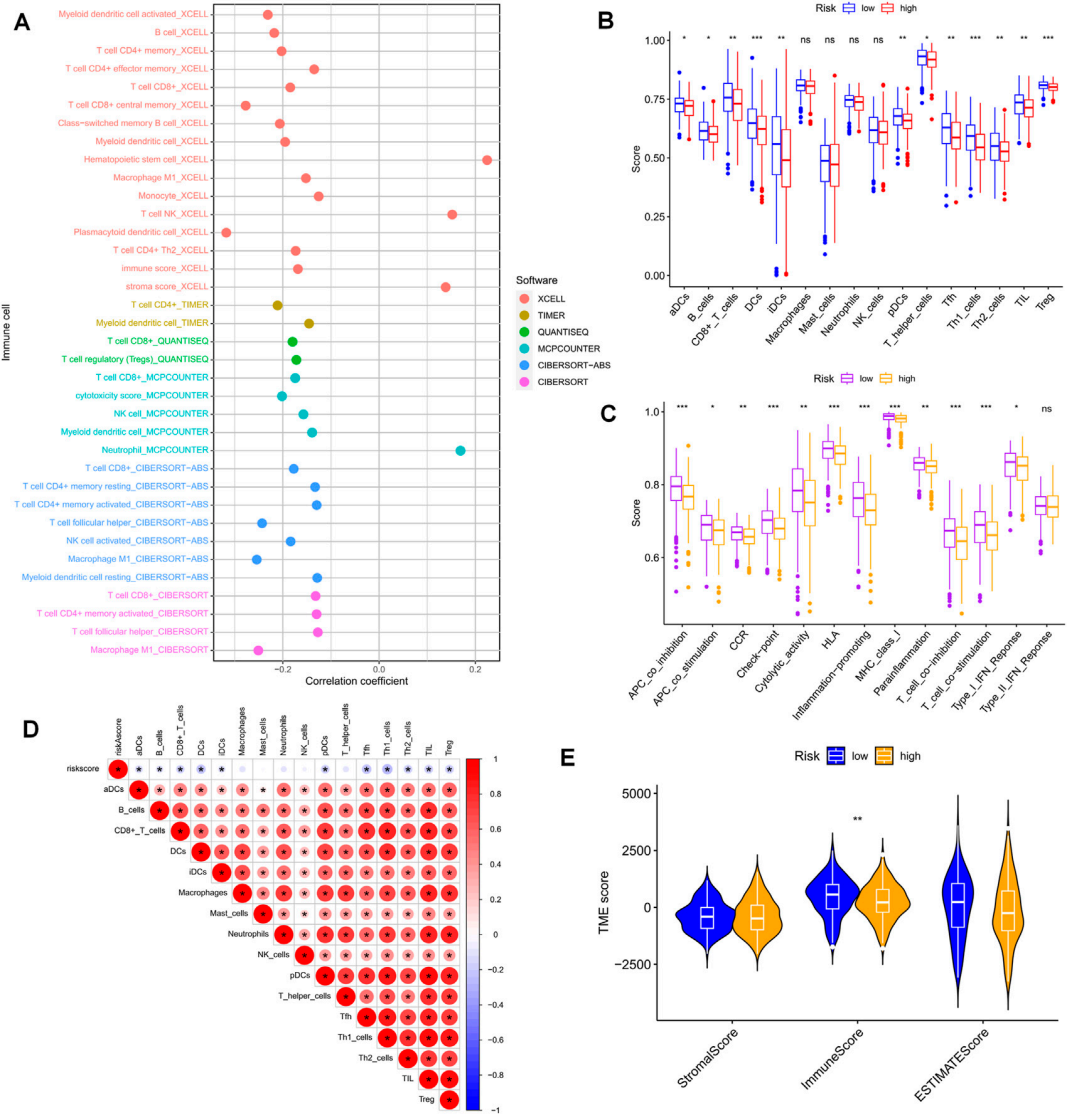
Risk score predicts TME and immune cell infiltration. **(A)** Bubble plots obtained by different algorithms show the correlation between risk scores and immune cell content. **(B)** Differences in immune cell infiltration between populations in different risk groups. **(C)** Differences in immune function between populations in different risk groups. **(D)** Correlation between immune cell infiltration scores and risk scores. **(E)** Differences in TME scores between populations in different risk groups. *p < 0.05, **p < 0.01, ***p < 0.001.

**FIGURE 9:**
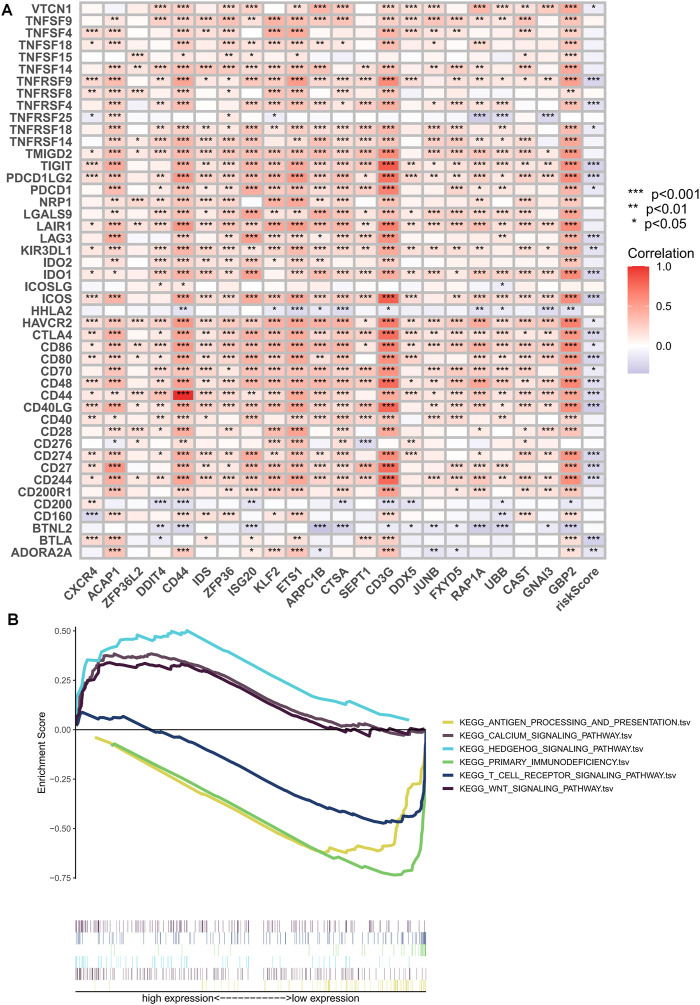
Immune checkpoint correlation analysis and GSEA enrichment analysis. **(A)** Correlation of expression of all immune checkpoints with model genes and risk scores. **(B)** GSEA analysis focused on the differential enrichment of KEGG pathways. *p < 0.05, **p < 0.01, ***p < 0.001.

The clinical outcome of patients and their response to treatment are significantly influenced by the tumor microenvironment (TME), which includes tumor-infiltrating immune cells (TIICs) that play a crucial role in tumorigenesis and progression (Low et al., 2022). We used algorithms from different platforms to investigate the correlation between risk scores and TIICs (Figure 8A). To gain deeper insights into the relationship between risk scores and immune cells and functions, we used the “ssGSEA” approach to measure the enrichment scores of various immune cell subpopulations, activities, or pathways (Figures 8B, C). The results indicated that the LR group had higher scores for immune-related functions and immune cell infiltration. Furthermore, the degree of immune cell infiltration showed a significant negative correlation with the risk score (Figure 8D). As immune checkpoint molecules have a profound influence on tumor immunotherapy, we investigated the correlation between risk scores and immune checkpoint (IC) expression. Notably, almost all immune checkpoint genes and our model genes displayed a strong correlation, including a significant positive correlation between CD3G and PD1, and CTLA4. Overall, our risk scores were negatively correlated with IC expression (Figure 9A). We used “ESTIMATE” to estimate tumor purity and calculate the proportion of stromal and immune cells in the different risk groups (Figure 8E). Collectively, these findings suggest that patients in the LR group have better prognoses, higher immunological activity, and possibly greater sensitivity to immunotherapy. To explore potential differences in biological function between the HR and LR groups, we performed a GSEA based on normalized enrichment scores (NES) and *p* values and identified the six most enriched signaling pathways (Figure 9B). Interestingly, lower risk scores were associated with T-cell receptors and immune-related signaling pathways, consistent with the theme of our study. Although our TEX-related prognostic model exhibited significant potential in identifying the immune landscape of patients and predicting their prognosis, we acknowledge limitations that need to be addressed in future studies, such as the use of more sophisticated techniques to detect TEX in the scRNA-seq data and validation of the model’s utility and accuracy in predicting immunotherapy outcomes using additional data from OC patients.

### Immunotherapy and drug sensitivity analysis

**FIGURE 10:**
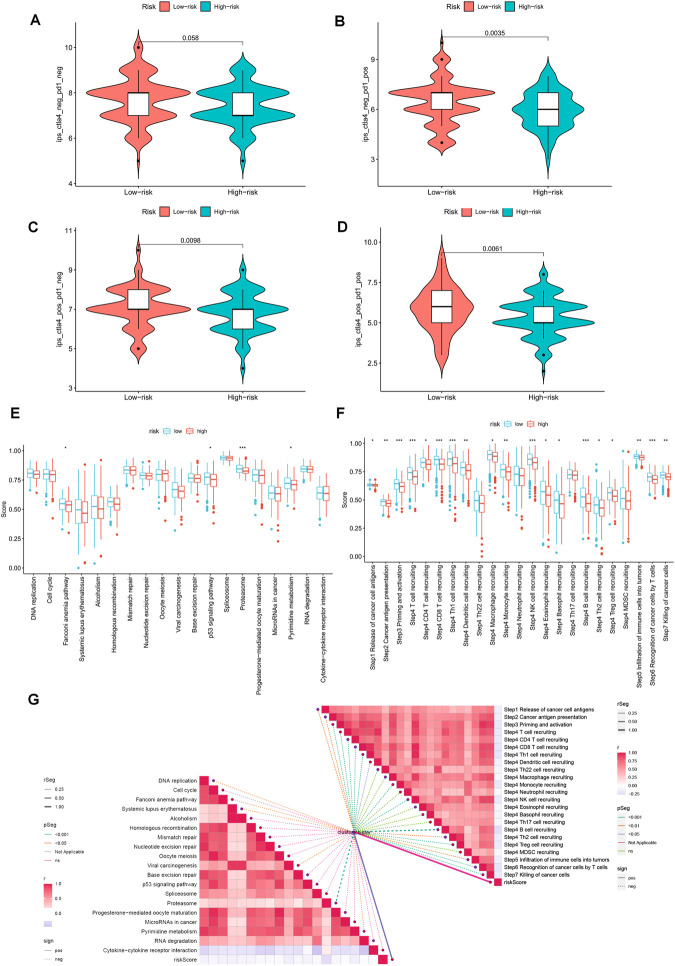
Prediction of immunotherapy for different risk subgroups. **(A–D)** Comparison of the relative distribution of Immunophenotype Score (IPS) between the HR and LR groups. **(E)** Differences in richness scores between the HR and LR groups on the predicted pathways for immunotherapy. **(F)** Differences between HR and LR groups at each step of the cancer-immune cycle. **(G)** Correlation of risk scores with ICB response characteristics and each step of the tumor-immune cycle. *p < 0.05, **p < 0.01, ***p < 0.001.

**FIGURE 11:**
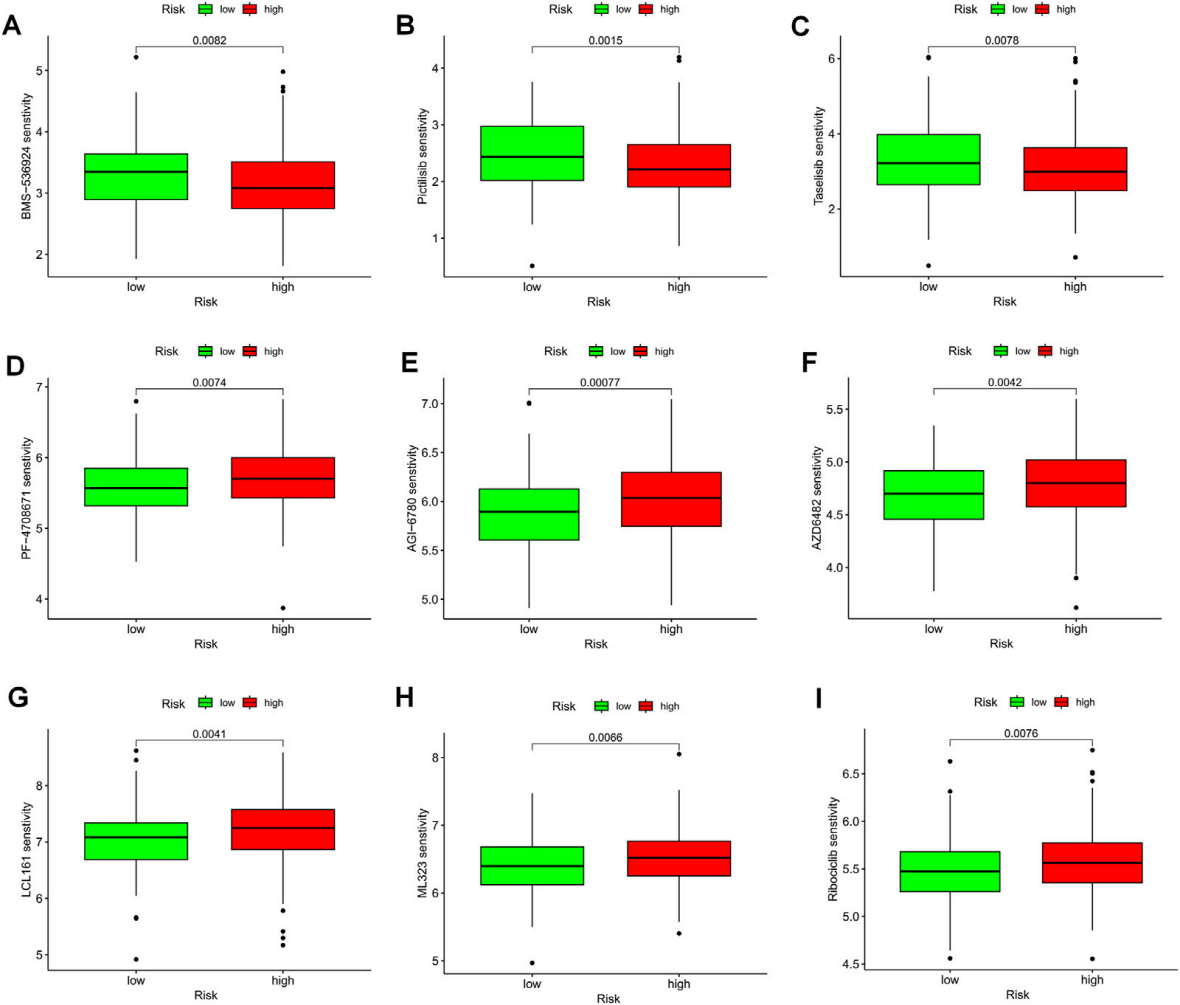
TEX-related prognostic signature predicted the sensitivity of chemotherapy. **(A)** BMS-536924, **(B)** Pictilisib, **(C)** Taselisib, **(D)** PF-4708671, **(E)** AGI-6780, **(F)** AZD6482, **(G)** LCL161, **(H)** ML323, and **(I)** Ribociclib.

The violin plot displayed in Figures 10A–D depicts the correlation between risk groups and the Immunophenotype Score (IPS), where higher IPS scores indicate better responses to PD-1 and CTLA-4 blockers. Immune checkpoint blockade (ICB) has been extensively studied as an immunotherapeutic agent that blocks inhibitory signaling of T-cell activation, thereby enabling tumor-reactive T cells to generate an effective anti-tumor response (Morad et al., 2021). Despite its significant advancements, ICB therapy only benefits a subset of patients. In order to investigate the association between risk scores and positive signals associated with ICB, we conducted a comprehensive analysis. The findings indicated that several signals, including the Proteasome, Fanconi anemia pathway, p53 signaling pathway, and Pyrimidine metabolism, exhibited an inverse correlation with risk scores (Figure 10G). Moreover, enrichment scores for these signals were higher in the LR group (Figure 10E). We also examined the biological functions of chemokine systems and immunomodulators and observed an increase in the activity of tumor immune steps among a subset of cycle steps in the LR group, including cancer antigen release (Step 1), cancer antigen presentation (Step 2), Priming and activation (Step 3), recruitment of immune cells (Step 4), infiltration of immune cells into the tumor (Step 5), and recognition of cancer cells by T cells (Step 6) (Figure 10F). Additionally, we discovered a significant negative correlation between each of these steps in the tumor immune cycle and risk score (Figure 10G). These findings suggest that patients in the LR group may be more responsive to ICB therapy. Furthermore, we investigated the correlation between risk scores and the IC50 of chemotherapeutic agents. As demonstrated in Figure 11, BMS-536924, Pictilisib, and Taselisib were found to be more effective in the LR group. Conversely, patients in the HR group were more responsive to PF-4708671, AGI-6780, AZD6482, LCL161, ML323, and Ribociclib.

### Validation of the expression of TEXRGs

**FIGURE 12:**
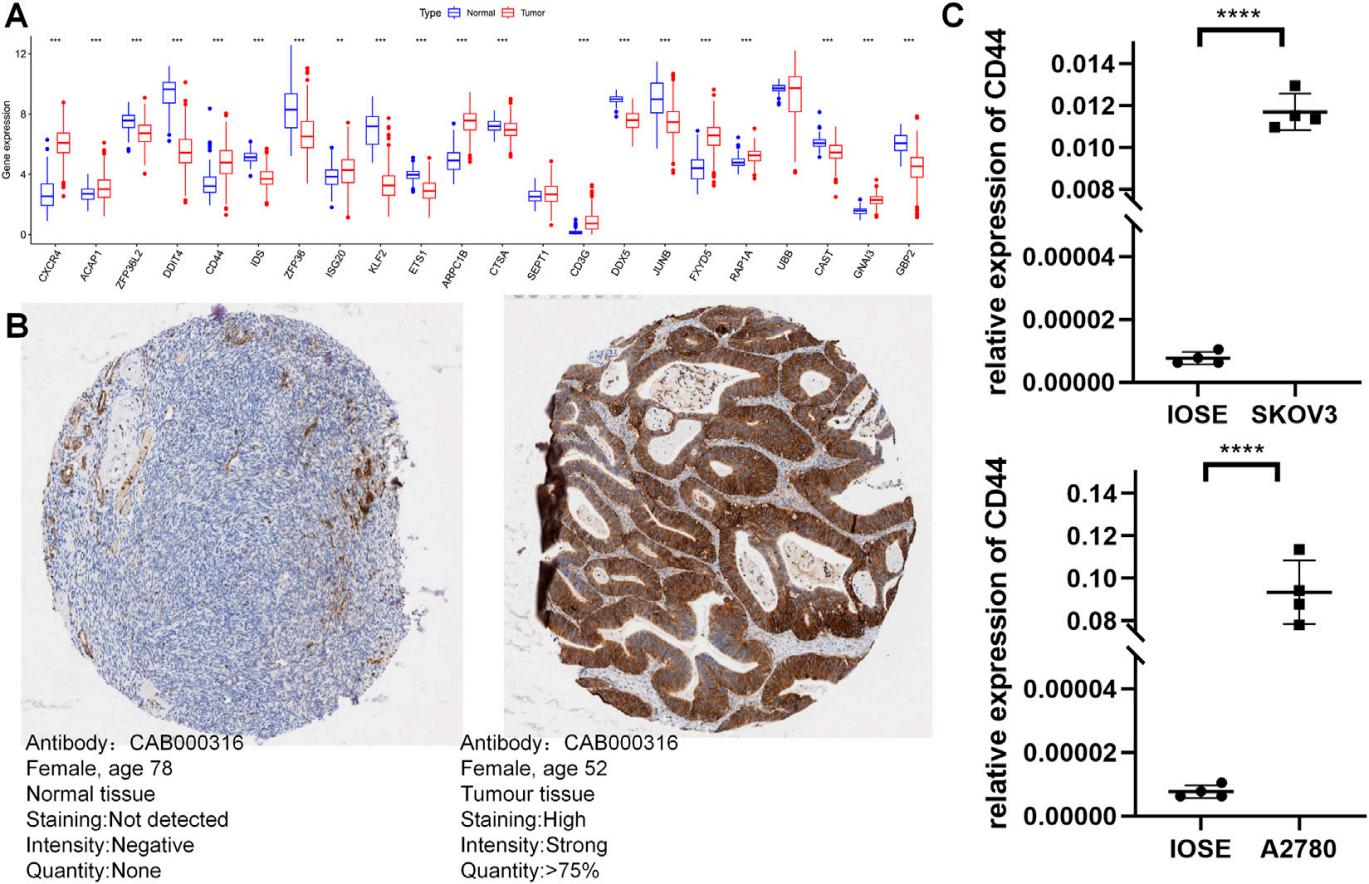
Validation of the expression of TEXRGs that constitute the risk model. **(A)** Differential expression of 22 model genes in normal and OC samples. **(B)** Immunohistochemical analysis of CD44 in normal ovarian tissue and ovarian cancer. **(C)** qRT-PCR analysis of CD44. **p < 0.01; ***p < 0.001; ****p < 0.0001.

The UCSC Xena database provided the combined TCGA-GTEx cohort, and expression levels of most model genes were differentially expressed between normal and tumor samples, except for SEPT1 and UBB (Figure 12A). We further confirmed the expression pattern of TEXRGs in OC patients using the HPA database for immunohistochemical data. We found that CD44 protein expression levels were markedly higher in ovarian cancer tissues compared to healthy liver tissues (Figure 12B). Additionally, qRT-PCR analysis demonstrated significantly upregulated CD44 expression levels in ovarian cancer cell lines (Figure 12C). These results suggest that abnormal expression of these genes may play a role in promoting oncogenic transformation in ovarian cancer.

## Discussion

Immunotherapy has shown promising results in treating tumors like melanoma and non-small cell lung cancer, particularly through the use of immune checkpoint inhibitor (ICB) therapy and peripatetic T lymphocyte therapy. However, the efficacy of immunotherapy for ovarian cancer is still being researched (Torre et al., 2018). Ovarian cancer is considered a “cool tumor,” with little infiltration of cytotoxic T lymphocytes (CTLs) and limited recognition of tumor antigens by infiltrating T lymphocytes (Cai and Jin, 2017; Zhao et al., 2023b). An important factor limiting the response to immunotherapy and disease progression in ovarian cancer is the immunosuppressed tumor microenvironment (Fialova et al., 2013). The immunosuppressive tumor microenvironment in ovarian cancer is a major obstacle to effective immunotherapy, as high levels of immunosuppressive molecules like VEGF and IL-10 induce regulatory T Cell differentiation and further inhibit the immune response of effector T lymphocytes against the tumor (Metz et al., 1975).

NFAT, a transcription factor activated by prolonged T Cell receptor (TCR) stimulation and downstream Ca2+ signaling, upregulates the transcription of suppressive immune checkpoint proteins such as PD-1, CTLA-4, CD-39, and LAG-3 in T cells (Liu et al., 2019; Scott et al., 2019; Seo et al., 2019). Combined receptor inhibitor therapy can block these signals, reverse tumor immune evasion, restore the function of tumor-infiltrating lymphocytes, and enhance the efficacy of immunotherapy (Dolina et al., 2021). These advances in ICB research and the understanding of the tumor immunosuppressive microenvironment have generated considerable interest in TEX.

The continuous phenotype and intermediate functional state known as TEX represent a persistent T-cell grade dysfunction that is increasingly recognized (Ma et al., 2019; Zhang et al., 2022). Understanding the dysregulated and diminished state of T cells in ovarian cancer is critical to overcoming the TEX barrier and enhancing the effectiveness of immune checkpoint blockade therapy. However, despite the significant role of T-cell exhaustion in the progression of several cancers, including ovarian cancer (OC), research on this topic remains limited. Hence, we aimed to develop a multi-biomarker model based on TEX-related genes to aid healthcare professionals in assessing the prognosis and tumor microenvironment of OC patients, as well as to establish a theoretical foundation for personalized precision therapy.

We performed clustering analysis on scRNA-seq data from the GSE154600 dataset and identified 22 cell subpopulations. Through cell communication analysis, we discovered that the CXCL signaling pathway plays a crucial role in T and B cells. The importance of chemokines (CXCL) in the development of tertiary lymphoid structures (TLS) has been emphasized in several studies. CXCL13, for example, has been identified as a prognostic factor in ovarian cancer owing to its association with the number of tumor-infiltrating lymphocytes (Dai et al., 2021; Ukita et al., 2022). Abnormal chemokine distribution promotes the differentiation and infiltration of immunosuppressive cells (e.g., Treg cells, MDSC, and TAM) into tumors (Bule et al., 2021). To identify the driver genes of TEX progression in ovarian cancer, we extracted T Cell marker genes and used the GSVA algorithm with TCGA-OC data to identify the most relevant key modules. We obtained a set of 185 post-selected genes by combining two GEO validation sets. Instead of using the Lasso method to model multiple marker genes, we used a metric that aggregated the expressions of these genes to construct the final model (Xie et al., 2022a; Xie et al., 2022b). Although the Lasso method aims to minimize the number of variables by compressing the coefficients of the variables and setting certain regression coefficients to zero through a penalty function, it has inherent drawbacks, such as not yielding an explicit solution and the estimated results being less stable and prone to error. To address these limitations, we transformed ten machine-learning algorithms into eighty combinations and developed a stable and robust TEX-related prognostic signature based on the average C-index of three ovarian cancer cohorts (Liu et al., 2022). We determined that the combination of CoxBoost and RSF was the optimal method to construct a novel and enduring prognostic model. Our analysis showed that the TEXRPS we developed was a robust predictor of prognosis in OC, and we identified significant differences in prognostic outcomes between the two groups. The predictive ability of TEX features for patient prognosis was demonstrated by ROC and calibration curve analyses, with excellent results (insert ROC curve and calibration curve images here). In addition, our nomograms highlighted the superiority of TEX-related prognostic features compared to various indicators currently utilized in clinical practice.

In our study, we have identified a panel of 22 genes that together serve as a stable risk score signature for ovarian cancer. Differential analysis revealed that 20 of these genes exhibit differential expression between tumor and normal tissues. Subsequently, we narrowed our focus to CD44 and CD3G for further experimental validation. The non-kinase transmembrane receptor CD44 represents a promising therapeutic target for ovarian cancer treatment, as it has been implicated in the development of chemoresistance, maintenance of cancer stem cells, and promotion of metastatic progression through diverse mechanisms (Martincuks et al., 2020). CD44 also facilitates tumor angiogenesis, immunosuppression, and metabolic reprogramming through interactions with STAT3, thereby supporting the pro-tumorigenic tumor microenvironment (Wielenga et al., 1993; Ponta et al., 2003; Li et al., 2014). Conversely, the CD3G protein, which forms the TCR/CD3 complex and is expressed on the surface of T cells, exhibits potent antitumor activity by recognizing tumor-associated antigens and initiating intracellular signaling (Marshall et al., 2018). Notably, CD3G-deficient patients display reduced T-cell diversity, diminished suppressor function, and increased autoimmune clonality, highlighting the potential of CD3G as a target for immunotherapy (Rowe et al., 2018; Wang et al., 2022). Nonetheless, the precise role of CD3G in ovarian cancer warrants further investigation.

The interaction between PD-L1 and PD-1 leads to the apoptosis and exhaustion of T lymphocytes, as previously reported (Chen and Han, 2015). Our findings are in line with previous studies, indicating that ovarian cancer patients with highly infiltrated PD-1-positive immune cells have a better prognosis (Ojalvo et al., 2018). Clinical trials of anti-PD-1 antibodies and anti-PD-L1 antibodies are currently underway, and early results suggest that anti-PD-1 antibodies alone may be effective in treating recurrent ovarian cancer, with PD-L1 expression strongly correlated with the efficacy of immune checkpoint blockade (ICB) therapy (Dai et al., 2018). It is widely accepted that the tumor microenvironment plays a critical role in various tumor phenotypes. In particular, immune cell infiltration, as a major feature of the tumor microenvironment, contributes significantly to the immune escape of tumor cells and the development of an inflammatory milieu (Tower et al., 2019). Therefore, understanding the immune cell infiltration and the features of Tumor-Associated Lymphocytes (TALs) in patients with ovarian cancer, stratified by different risk groups, can provide valuable insights into the overall immune status of patients and the role of immune regulation in tumor development. Immunotherapy has been explored in ovarian cancer; however, its efficacy remains limited, possibly due to the tumor’s heterogeneity, lack of antigenic targets, and low infiltration of immune cells in the ovarian tumor microenvironment. Tumor-Associated Exosomes (TEX) express immunosuppressive receptors, and immune checkpoint inhibitors can counteract these signals, reverse TEX, and restore TAL function. Combining ICB with immune checkpoint inhibitors can partially overcome the adaptive immune resistance in the tumor microenvironment, thus restoring the immune function of CD8^+^ T lymphocytes in the tumor microenvironment and enhancing the anti-tumor immune response (Pan et al., 2022).

Although our TEX-related prognostic features have demonstrated remarkable ability in identifying the immune landscape of patients and predicting their prognosis, there are still limitations that must be acknowledged and addressed in future studies. Firstly, due to the limited conditions and number of cells, our scRNA-seq data was insufficient to detect TEX, and more sophisticated techniques and tools are required to construct models. Furthermore, analyzing data from public databases may introduce bias in predictions that do not reflect the actual situation. Therefore, further data from OC patients are necessary to validate the model’s utility and accuracy in predicting immunotherapy outcomes.

In conclusion, our findings suggest that the TEX-related prognostic signature is a unique and promising prognostic biomarker and therapeutic target for OC patients. The TEXRPS enables the characterization of the immune microenvironment of OC patients and the accurate prediction of their prognosis, thus facilitating the identification of patient subgroups that may benefit from personalized treatment with immunotherapy and chemotherapy.

## Data Availability

Publicly available datasets were analyzed in this study. This data can be found here: https://www.jianguoyun.com/p/DfRmD9wQ0pH7Chjv_P4EIAA.
