# Supplementary figures and images for: A novel T-cell exhaustion-related feature can accurately predict the prognosis of OC patients
Source: Front Pharmacol. 2023 May 22;14:1192777. doi: 10.3389/fphar.2023.1192777 (PMC10239809; doi:10.3389/fphar.2023.1192777)

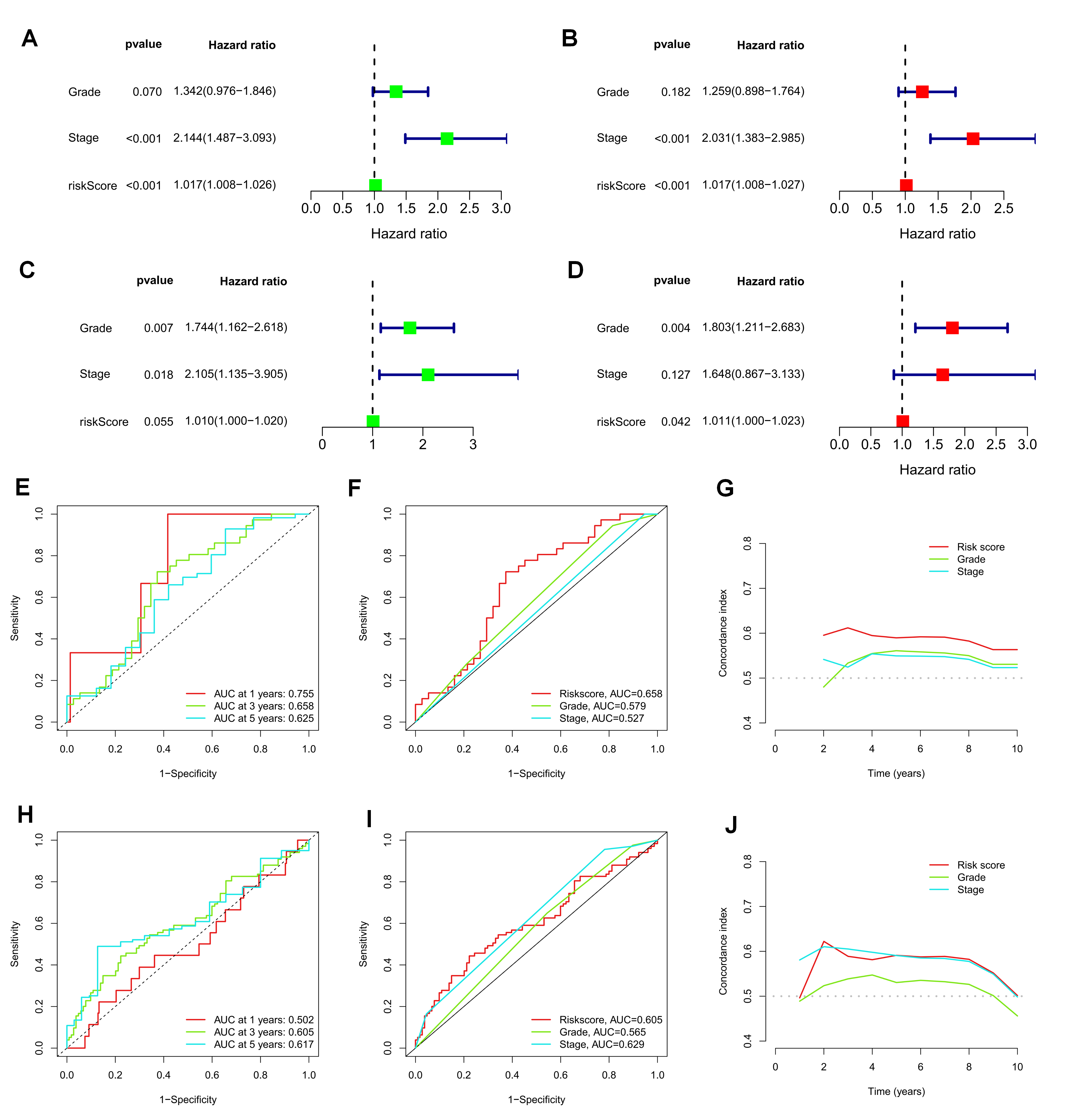

Supplement: Supplementary file 3 [file Image3.TIF]

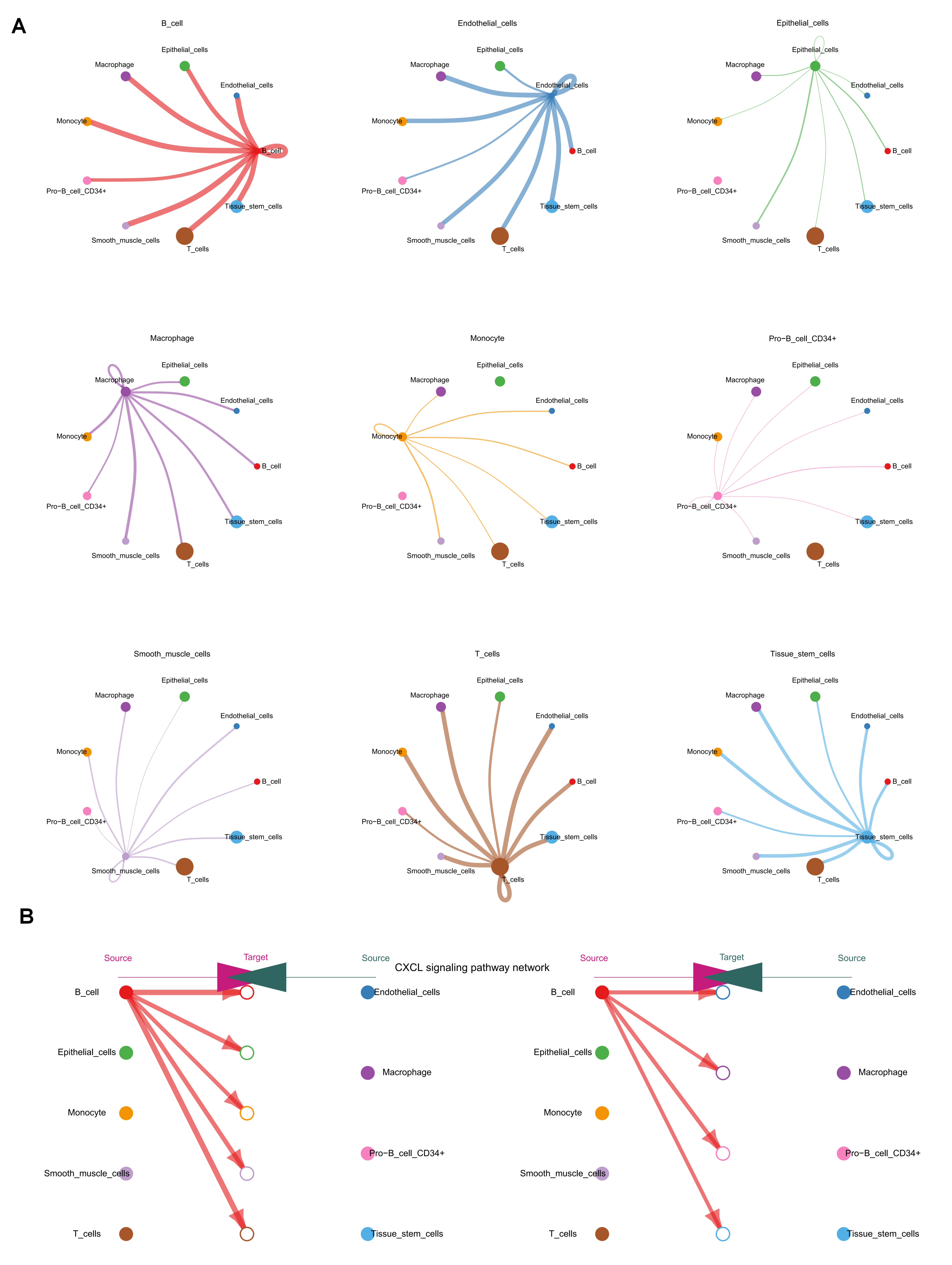

Supplement: Supplementary file 4 [file Image2.TIF]

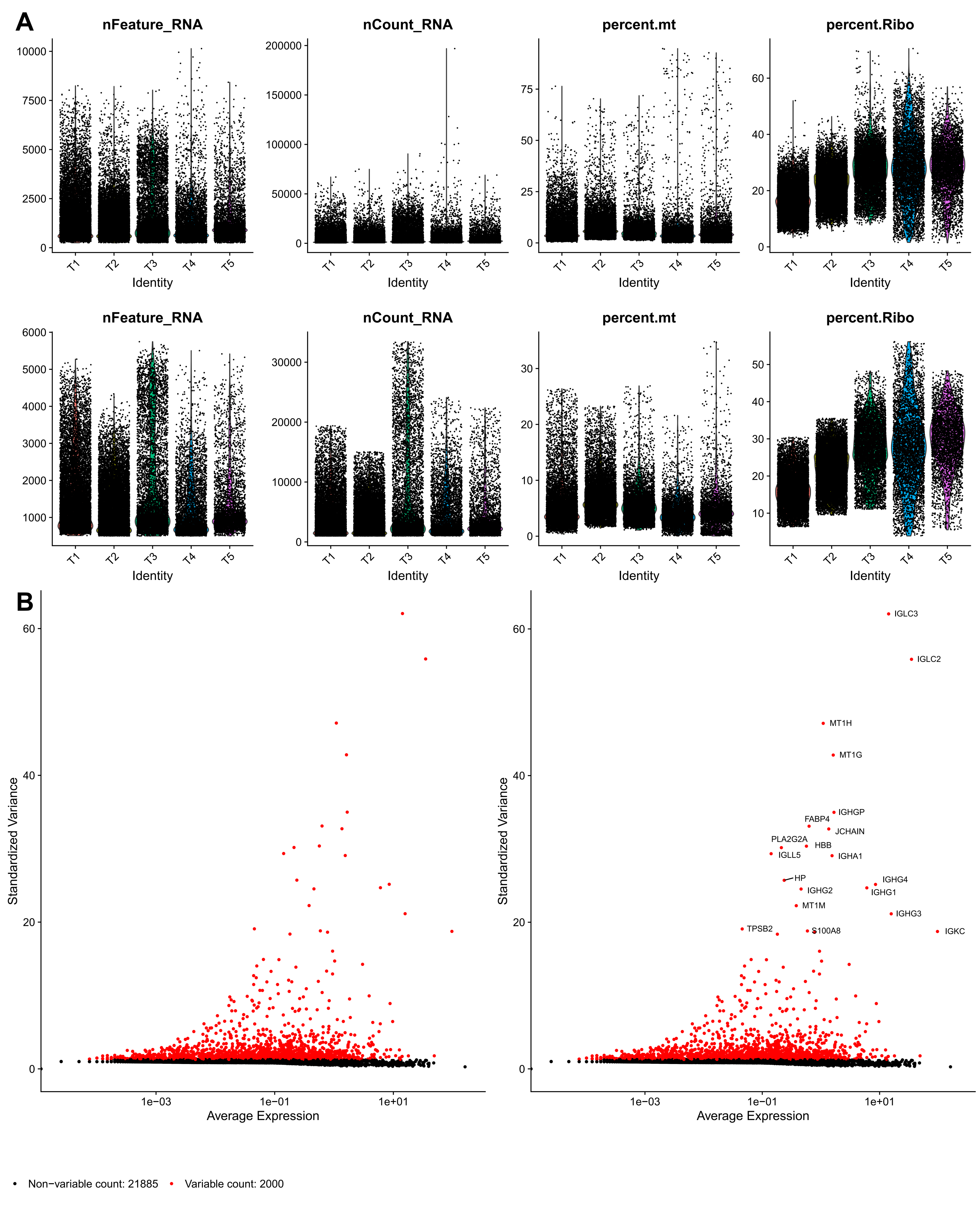

Supplement: Supplementary file 5 [file Image1.TIF]
